# Diagnosis of Dizziness Due to a Core Vestibular Projection Injury in a Patient with Intracerebral Hemorrhage

**DOI:** 10.3390/diagnostics10040220

**Published:** 2020-04-15

**Authors:** Hyeok Gyu Kwon, Chul Hoon Chang, Sung Ho Jang

**Affiliations:** 1 Department of Physical Therapy, College of Health Science, Eulji University, Sungnam 13135, Korea; khg0715@hanmail.net; 2 Department of Neurosurgery, College of Medicine Yeungnam University, Daegu 42415, Korea; cch0102@yumail.ac.kr; 3 Department of Physical Medicine and Rehabilitation, College of Medicine, Yeungnam University, Daegu 42415, Korea

**Keywords:** core vestibular projection, dizziness, diffusion tensor imaging, diffusion tensor tractography, intracerebral hemorrhage, parieto-insular vestibular cortex

## Abstract

Herein, we present a patient diagnosed with dizziness due to a core vestibular projection injury following intracerebral hemorrhage (ICH). A 51-year-old female patient underwent conservative management for a spontaneous ICH in the left hemisphere (mainly affecting the basal ganglia and insular cortex). When she visited the rehabilitation department of the university hospital at two years after the ICH onset, she advised of the presence of moderate dizziness (mainly, light-headedness) that started after ICH onset. She mentioned that her dizziness had decreased slightly over time. No abnormality was observed in the vestibular system of either ear on physical examination by an otorhinolaryngologist. However, diffusion tensor tractography results showed that the core vestibular projection in the left hemisphere was discontinued at the basal ganglia level compared with the patient’s right core vestibular projection and that of a normal subject. Therefore, it appears that the dizziness in this patient can be ascribed to a left core vestibular projection injury.

**Figure 1:**
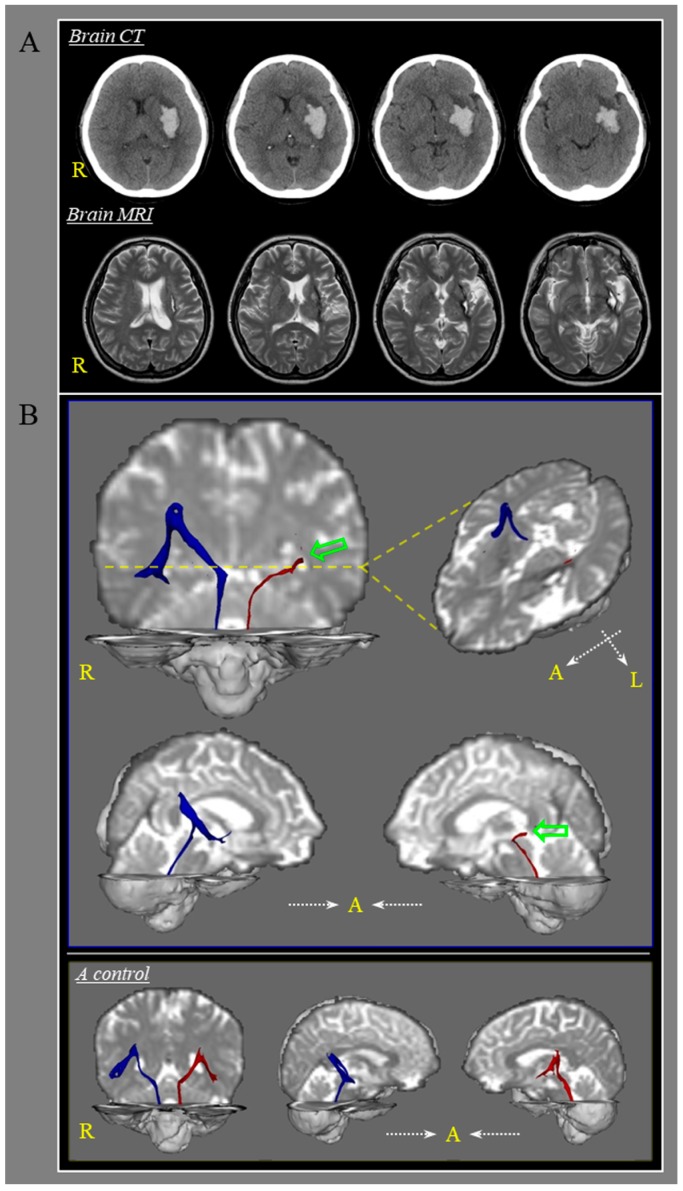
A 51-year-old female patient underwent conservative management for a spontaneous intracerebral hemorrhage (ICH) in the left basal ganglia (mainly affecting the internal capsule and insular cortex) (**A**). When she visited the rehabilitation department of the university hospital at two years after the ICH onset, she reported that moderate dizziness (mainly, light-headedness) had started after the ICH onset. She mentioned that her dizziness had decreased slightly over time. Physical examination by an otorhinolaryngologist revealed no abnormalities in the vestibular system of either ear. She also exhibited mild right hemiparesis; however, her cognitive function was within the normal range (Mini-Mental State Examination score, 28, cutoff value: <25). Brain magnetic resonance images were obtained two years after onset and revealed leukomalactic lesions in the left basal ganglia and the subcortical white matter of the insular cortex (**A**). On diffusion tensor tractography, the patient’s core vestibular projection in the left hemisphere (red) showed discontinuation (green arrows) at the basal ganglia level compared with that of the patient’s right core vestibular projection (blue) and those of a normal subject (**B**). The patient and a normal subject (50-year-old female) provided written informed consent for participation in this study, and the study protocol was approved by the Yeungnam university hospital institutional review board (YUMC-2019-06-032, 28 June 2019). A: anterior, R: right, L: left, Yellow dotted line: level of lesion.

Diffusion tensor imaging data were acquired two years after the intracerebral hemorrhage (ICH) onset by using a six-channel sensitivity encoding head coil on a 1.5 T Philips Gyroscan Intera (Hoffman-LaRoche, Best, Netherlands) scanner. For each of the 32 noncollinear diffusion-sensitizing gradients, 67 contiguous slices were acquired parallel to the anterior commissure–posterior commissure line. Imaging parameters were as follows: acquisition matrix = 96 × 96; reconstructed to matrix = 192 × 192; field of view = 240 mm × 240 mm; repetition time = 10,398 msec; echo time = 72 msec; parallel imaging reduction factor = 2; echo-planar imaging factor = 59; b = 1000 sec/mm^2^; and slice thickness = 2.5 mm. Head motion effects and image distortion due to eddy currents were corrected by applying affine multiscale two-dimensional registration. Fiber tracking was performed by using probabilistic tractography as applied in the default tractography option of the diffusion software of the Oxford Centre for Functional Magnetic Resonance Imaging of the Brain [1]. 

For an imaging-based reconstruction of the core vestibular projection, the seed region of interest was placed on the vestibular nuclei that corresponded to Schwalbe’s and Deiters’ nuclei at the pons level [2,3,4]. The target region of interest was positioned at the parieto-insular vestibular cortex (PIVC) [2,3,4]. The core vestibular projection passes from the vestibular nuclei at the level of the pons to the PIVC via the posterolateral thalamus [2]. The PIVC, first described by Pandya and Sanides (1973), is located in the depth of the Sylvian fissure region, corresponding to the posterior parietal operculum/retroinsular region, and extends into the posterior part of the insular lobe [5,6]. The PIVC is reported to be involved in processing self-motion perceptions and suppressing visual motion stimulation, particularly motion related to the effects of gravity [7]. Therefore, the core vestibular projection has a key role in processing motion perception and spatial orientation. On diffusion tensor tractography (DTT), the patient’s core vestibular projection in the left hemisphere showed discontinuation at the basal ganglia level compared with that of the patient’s right core vestibular projection and those of a normal subject (B). Therefore, it appears that the dizziness in this patient may be ascribed, at least in part, to an injury of the left core vestibular projection [8,9]. However, our method for DTT should be considered as it is based on a large b-value and gradients and there can be problems with systematic errors, which can be most fully addressed in the Generalized Stejskal–Tanner equation [10,11,12]. Therefore, further studies to overcome the above limitation should be encouraged.

Two studies using DTT have reported on core vestibular projection injuries following a cerebral infarct [4,13]. In 2017, Yeo et al. demonstrated that core vestibular projection injuries were associated with central vestibular disorders in 19 patients with middle cerebral artery territory infarctions [4]. The following year, Yeo et al. [2018] reported on patients with dizziness due to core vestibular projection injuries following lateral medullary infarctions [13]. The present case study is the first to demonstrate the relationship between dizziness and a core vestibular projection injury in ICH. The results indicate that the application of DTT to the core vestibular projection would be useful in patients exhibiting dizziness following an ICH in the PVIC region. However, because it is based on a case report, this study is limited. Therefore, further studies involving a large number of subjects should be warranted.
